# Supplementary material for: Content-rich biological network constructed by mining PubMed abstracts
Source: BMC Bioinformatics. 2004 Oct 8;5:147. doi: 10.1186/1471-2105-5-147 (PMC528731; doi:10.1186/1471-2105-5-147)
Supplement: Additional File 2 — The original results of the above study (non-essential files are deleted to keep the file size under the limit set by BMC bioinformatics). [file 1471-2105-5-147-S2.bz2 › chilibotAdditionalFile2/dip30/94ID9178909E401/html/CBL_CRK.html]

 


 **CBL** and **CRK** 
  
Found 57 abstracts in PubMed, retrieved 30.  
 

 What does Google say? 
 PDF only 
| .edu only 

---

**Interactive relationship** (e.g. stimulation, inhibition, etc)

**Stimulatory relationship**- **Cbl**  b constitutively binds Grb2 and becomes associated with  **Crk**  L upon TCR stimulation.  Ref: 10022120 Oncogene, 1999
- Like c  **Cbl** ,  **Cbl**  b associates constitutively with CAP and interacts with  **Crk**  upon insulin stimulation.  Ref: 12842890 J Biol Chem< MedlineTA>J Biol Chem, 1999
- c  **Cbl**  and Nck are also tyrosine phosphorylated by cellular stimulation with hGH and associate with the p130 Cas CrkII  [ **CRK** ]  complex.  Ref: 9837978 J Biol Chem, 1998
- We also show that both  **Cbl**  and Gab1 enhance Met induced activation of another MAP kinase cascade, the ERK pathway, in a  **Crk**  independent manner.  Ref: 10962563 Oncogene, 2000
- **Cbl**  is rapidly tyrosine phosphorylated in response to stimulation of a variety of cell surface receptors and becomes associated with a number of intracellular signalling molecules such as protein tyrosine kinases, phosphatidylinositol 3 kinase,  **Crk** , and 14 3 3 proteins through different protein interacting modules, leading to the formation of multimolecular signalling complexes.  Ref: 9720760 Cell Signal, 1998
**Neutral relationship**- In  **Cbl**  b T cells, the interaction between  **Crk**  L and C3G, and the activity of the small GTPase Rap1 are increased.  Ref: 12697763 J Biol Chem< MedlineTA>J Biol Chem, 1998
- It was previously reported that  **Cbl**  b associates with  **Crk**  L in Jurkat T cells.  Ref: 12697763 J Biol Chem< MedlineTA>J Biol Chem, 1998
- In Met transformed cells,  **Crk**  predominantly associates with the  **Cbl**  and Gab1docking proteins in a tyrosine phosphorylation dependent manner.  Ref: 11146548 Oncogene, 2000
- Here we show that phosphorylated  **Cbl**  recruits the CrkII  [ **CRK** ]  C3G complex to lipid rafts, where C3G specifically activates the small GTP binding protein TC10.  Ref: 11309621 Nature, 2001
- Thus,  **Cbl**  b plays a negative role in  **Crk**  L C3G mediated Rap1 and LFA 1 activation in T cells.  Ref: 12697763 J Biol Chem< MedlineTA>J Biol Chem, 2001
- The Grb2 and the  **Crk**  L binding regions were mapped to the C terminus of  **Cbl**  b.  Ref: 10022120 Oncogene, 1999
- We found that  **Cbl**  b associates with, and induces ubiquitin conjugation to  **Crk**  L, which requires a functional RING finger.  Ref: 12697763 J Biol Chem< MedlineTA>J Biol Chem, 1999
- BCR engagement causes  **Crk**  to bind to the Cas and  **Cbl**  docking proteins.  Ref: 9786933 J Biol Chem, 1998
- Although the exact mechanism of interaction between these molecules has not been fully defined, it has been generally accepted that  **Crk** , ZAP 70, and p85 interact with tyrosine phosphorylated  **Cbl** , which serves as a major scaffold protein in activated T lymphocytes.  Ref: 11418612 J Biol Chem, 2001
- The proto oncogene c  **Cbl**   [ **CBL** ]  is a positive regulator of Met induced MAP kinase activation a role for the adaptor protein  **Crk** .  Ref: 10962563 Oncogene, 2000
- T cell activation induces direct binding of the  **Crk**  adapter protein to the regulatory subunit of phosphatidylinositol 3 kinase p85 via a complex mechanism involving the  **Cbl**  protein.  Ref: 11418612 J Biol Chem, 2001
- **Cbl**  serves as a substrate of both receptor and non receptor tyrosine kinases, and binds to adaptor proteins Grb2,  **Crk**  and the p85 subunit of PI 3 kinase.  Ref: 9675877 Int J Biochem Cell Biol, 1998
- The  **Crk**  adapter proteins are assumed to play a role in T lymphocyte activation because of their induced association with tyrosine phosphorylated proteins, such as ZAP 70 and  **Cbl** , and with the phosphatidylinositol 3kinase regulatory subunit, p85, following engagement of the T cell antigen receptor.  Ref: 11418612 J Biol Chem, 2001
- We report here that c  **Cbl** , which is a Gab1 like docking protein, also becomes tyrosine phosphorylated in response to Met activation and serves as a docking molecule for various SH2 containing molecules, including  **Crk** .  Ref: 10962563 Oncogene, 2000
- The overexpression of a CAP mutant in which the SoHo domain had been deleted CAPDeltaSoHo prevented the translocation of  **Cbl**  to lipid rafts and subsequently blocked the recruitment of CrkII  [ **CRK** ]  and C3G.  Ref: 11481476 Proc Natl Acad Sci U S A, 2001

**Non-interactive relationship** (e.g. studied together, co-existance, homology, etc.)

- Other signalling molecules are also clearly involved, including p62 DOK, p95 VAV,  **CRK**  L, p12O  **CBL**  and focal adhesion proteins.  Ref: 9844814 Hematol Cell Ther, 1998
- Overexpression of the APS Y 618 F mutant in 3T3 L1 adipocytes blocked the insulin stimulated tyrosine phosphorylation of endogenous  **Cbl**  and binding to  **Crk** .  Ref: 11997497 Mol Cell Biol, 2002
- Activation of nuclear factor of activated T cells NFAT and activating protein 1 AP 1 by oncogenic 70Z  **Cbl**  requires an intact phosphotyrosine binding domain but not  **Crk**  L or p85 phosphatidylinositol 3 kinase association.  Ref: 9988765 J Biol Chem, 1999
- This process is independent of PI 3 K, but requires the translocation of  **Cbl** ,  **Crk**  and C3G to the lipid raft.  Ref: 11309621 Nature, 2001
- It was found in complexes with many signaling proteins, including phosphoinositol PtdIns 3 kinase EC 2.7.1.137,  **Cbl** , GRB2, p130Cas and  **Crk** .  Ref: 12135478 Eur J Biochem, 2002
- This study therefore identifies a cytoskeletal role for c  **Cbl**  which may involve the regulation of  **Crk**  and Rac, and which is dependent on targeting of c  **Cbl**  to actin lamellae and the ability to recruit signalling protein s associated with its divergent SH2 domain.  Ref: 10633073 J Cell Sci, 2000
- We further show that  **Cbl**  is similarly capable of enhancing Met induced JNK activation, and several lines of experimentation suggests that it does so by interacting with  **Crk** .  Ref: 10962563 Oncogene, 2000
- When overexpressed in these cells, wild type APS but not an APS Y 618 F mutant facilitated the tyrosine phosphorylation of coexpressed  **Cbl**  and its association with  **Crk**  upon insulin stimulation.  Ref: 11997497 Mol Cell Biol, 2002
- We conclude that 70Z  **Cbl**  induced NFAT AP1 activation requires the phosphotyrosine binding domain but not  **Crk**  L p85 PI3K association.  Ref: 9988765 J Biol Chem, 1999
- In addition, SDF 1alpha stimulation enhanced the association of  **cbl**  with PI 3 kinase,  **Crk**  L, and 14 3 3beta proteins.  Ref: 11157475 Blood, 2001
- We demonstrate here that PKA s activation of Rap1 was mediated by the Rap1 guanine nucleotide exchange factor C3G, the adaptor  **Crk**  L, the scaffold protein  **Cbl** , and the tyrosine kinase Src.  Ref: 11804588 Mol Cell, 2002
- EphA activation also induced  **Cbl**  association with  **Crk**  L and  **Crk**  II  [ **CRK** ]  adapters, but not the related Grb2 protein.  Ref: 12794130 J Immunol, 2003
- Here we show that, like TCR CD3 stimulation, stimulation of CD2 enhanced the association of c  **Cbl**  with both  **Crk**  L and the p85 subunit of phosphatidylinositol 3 kinase.  Ref: 11133830 Int Immunol, 2001
- Here we examined a regulatory role of  **Cbl**  b in  **Crk**  L C3G signaling pathway.  Ref: 12697763 J Biol Chem< MedlineTA>J Biol Chem, 2001
- We demonstrate that 70Z  **Cbl**  shows increased basal and activation induced tyrosine phosphorylation and association with  **Crk**  L and p85 PI3K in Jurkat T cells.  Ref: 9988765 J Biol Chem, 1999
- However, unlike in other cell lines, CrkII  [ **CRK** ]  was not inducibly bound to  **Cbl**  in U87MG glioma cells.  Ref: 10595738 Jpn J Cancer Res, 1999
- We find that Rap1 and proposed regulators of Rap1 the proto oncogene product  **Cbl** ,  **Crk**  family adaptor proteins, and the Rap1 guanine nucleotide exchange factor C3G are expressed at equivalent levels in both double negative and double positive murine thymocytes.  Ref: 11069064 Eur J Immunol, 2000
- The coupling of Gab1, but not  **Cbl** , with  **Crk**  is retained in cells grown in suspension and enhances JNK activation.  Ref: 11146548 Oncogene, 2000
- There is an activation induced association of tyrosine phosphorylated  **Cbl**  with Grb2 and CrkL, respectively, but not CrkII  [ **CRK** ] .  Ref: 11263968 Biochem Biophys Res Commun, 2001
- Negative regulation of TCR mediated  **Crk**  L C3G signaling and cell adhesion by  **Cbl**  b.  Ref: 12697763 J Biol Chem< MedlineTA>J Biol Chem, 2001
- 70Z  **Cbl**  retains the ability to enhance NFAT and activating protein 1 AP1 activity in the absence of  **Crk**  L p85 PI3K association.  Ref: 9988765 J Biol Chem, 1999
- By immunofluorescence microscopy we have determined that c  **Cbl**  co localizes with the adaptor protein  **Crk**  to submembranous actin lamellae in NIH 3T3 fibroblasts.  Ref: 10633073 J Cell Sci, 2000
- **Cbl**  b deficiency does not affect  **Crk**  L stability, but its association with C3G.  Ref: 12697763 J Biol Chem< MedlineTA>J Biol Chem, 2000
